# Introduction to serial reviews: Oxidative stress and digestive diseases

**DOI:** 10.3164/jcbn.17-intro

**Published:** 2018-04-11

**Authors:** Hidekazu Suzuki

**Affiliations:** 1 Fellowship Training Center and Medical Education Center, Keio University School of Medicine, 35 Shinanomachi, Shinjuku-ku, Tokyo 160-8582, Japan

The digestive tract is always in contact with the outside world and is an organ that is constantly exposed to foreign antigens because of feeding behavior^(1)^ and continuous exposure to micro-organisms.^(2,3)^ Furthermore, because the liver is a large-scale metabolic factory of ingested nutrients and drugs, it is also easily exposed to oxidative stress because extensive oxygen is always used in the process of metabolism.^(4,5)^ Oxidative stress in the pathogenesis of digestive diseases is an important theme that has been discussed for a long time.^(6,7)^ Inflammation and carcinogenesis, which are dual characteristics of the pathology of the digestive organs, also always involve this concept of oxidative stress. The aim of this serial review was to promote researchers’ and clinicians’ understanding of the oxidative aspects of the pathogenesis of digestive diseases and to foster research activities in the fields of gastroenterology and hepatology by cultivating the novel approaches or ideas to the oxidative stress-related disease process. In this serial review, I invite several outstanding researchers in this field to summarize their work and to review their peers’ scientific work, and to organize this knowledge according to their opinions. I thank all the anonymous and volunteer reviewers of these articles for their excellent insights.
